# Utilization of traditional Chinese medicine in the intensive care unit

**DOI:** 10.1186/s13020-021-00496-1

**Published:** 2021-08-23

**Authors:** Xia Zhang, Mingqi Wang, Wen Wang, Ling Li, Xin Sun

**Affiliations:** 1 Chinese Evidence-Based Medicine Center, West China Hospital, Sichuan University, 37 Guo Xue Xiang, Chengdu, 610041 Sichuan China; 2 NMPA Key Laboratory for Real World Data Research and Evaluation in Hainan, Chengdu, China; 3 Sichuan Center of Technology Innovation for Real World Data, Chengdu, China

**Keywords:** Traditional Chinese medicine, Intensive care unit, Utilization

## Abstract

Previous studies showed that traditional Chinese medicine (TCM) may be effective for patients with critical conditions. However, the extent to which TCM is used in intensive care units (ICUs) remains less investigated. This study aimed to investigate the utilization of TCM among ICU patients. Using a cross-sectional study design, we examined the use of TCMs among ICU patients. The data were from a well-established ICU registry from a large teaching hospital in west China. Our study found that TCM was widely used among ICU, in particular TCM injections and oral liquids. The use of TCM often differed by ICUs, and TCM injections and oral liquids were mostly used in neurological ICU, while Chinese herbal medicines (CHMs) were often used in general ICU. The use of TCM was also highly associated with patient characteristics. Patients with pancreatitis were administered with most TCM, patients with cerebrovascular disease with most TCM injections and those with chronic renal failure with most oral liquids. Future efforts should include generation of high-quality evidence guidelines about clinical effects of TCM interventions among ICU patients.

**Supplementary Information:**

The online version contains supplementary material available at 10.1186/s13020-021-00496-1.

## Background

Traditional Chinese medicine (TCM) is one of the most popular complementary and alternative medicine modalities worldwide [1]. Chinese governmental authorities have attached high importance to the development TCM and have issued 26 policies and measures from 2012 to 2019 [2]. These efforts were even strengthened in the past 2 years. As one of its targeted uses, TCM is often used among patients with critical conditions such as ischemic heart diseases and coronavirus disease 2019 (COVID-19) [3, 4]. Previous systematic reviews also suggested that TCM may be effective managing these patients [5–8]. However, the extent to which TCM is used among patients with critical conditions, particularly those at intensive care units (ICUs), is less investigated. The lack of clear understanding about the use of TCM has prevented better characterization of clinical values of TCM among these patients. Therefore, using a cross-sectional study, we systematically investigated the pattern of using TCM among patients at ICUs.

## Main text

This study was conducted using data from a large registry of 22,343 ICU patients from the West China Hospital (WCH), Sichuan University, a leading medical center in west China. The registry integrated multiple database systems, including ICU data, healthcare-associated infections at ICUs (ICU-HAI) and electronic medical records (EMRs). Details of this registry were published elsewhere and data in the registry had a high level of completeness and accuracy [9–11].

In this study, we included patients who were admitted to ICUs from April 1, 2015 to December 31, 2018. Patients were excluded if they met any of the following: younger than 18 years old; lack of critical information (e.g., date of birth, discharge diagnosis); and abnormal costs or length of stay at ICU (i.e., total cost equal to 0 or length of stay > 365 days). Patient characteristics were extracted from the registry, including demographics, hospitalization at ICUs (admission and discharge date, ICU wards); prescription (drug names, drug types, dose, route, date of prescriptions); and diagnostic information (outpatient, admitting and discharge diagnosis). Patient comorbidities were identified according to the International Classification of Diseases, 10th edition (ICD-10). The completeness and accuracy of the ICD-10 were 99% and 88%, respectively [9]. The information about TCM use was collected from ICU prescriptions which were documented in the EMRs. We established drug dictionary using drug codes provided by the hospital, which were previously validated [12] and contained information regarding drug name, route of administration, usage, and type of TCM.

Of the 22,343 patients, 6583 (29.5%) were prescribed with TCM, including 5424 (24.3%) using TCM injection, 1740 (7.8%) using TCM oral liquid and 790 (3.5%) using Chinese herbal medicine (CHM) (Additional file 1: Table S1). A total of 1881 patients were treated with multiple TCM injections and oral liquids, of whom 1336 (71.1%) were treated with multiple TCM injections, including 1025 (77.8%) receiving two TCM injections and 311 (55.2%) receiving more than two TCM injections (Additional file 1: Table S2).

A total of 438 TCMs were used in the ICU. Among all TCM used, Bupleurum injection (13.5%), Myrtol standardized enteric capsules (3.0%) and Jiang Magnolia (3.3%) were the most used TCM injection, oral liquid and CHM (Additional file 1: Table S3). The use of TCM prescriptions also differed by ICUs. While TCM injections and oral liquids were most frequently used in the neurological ICU (53.1% and 13.5%, respectively), and CHM were often used in the general ICU (7.2%) (Additional file 1: Table S4).

**Fig. 1:**
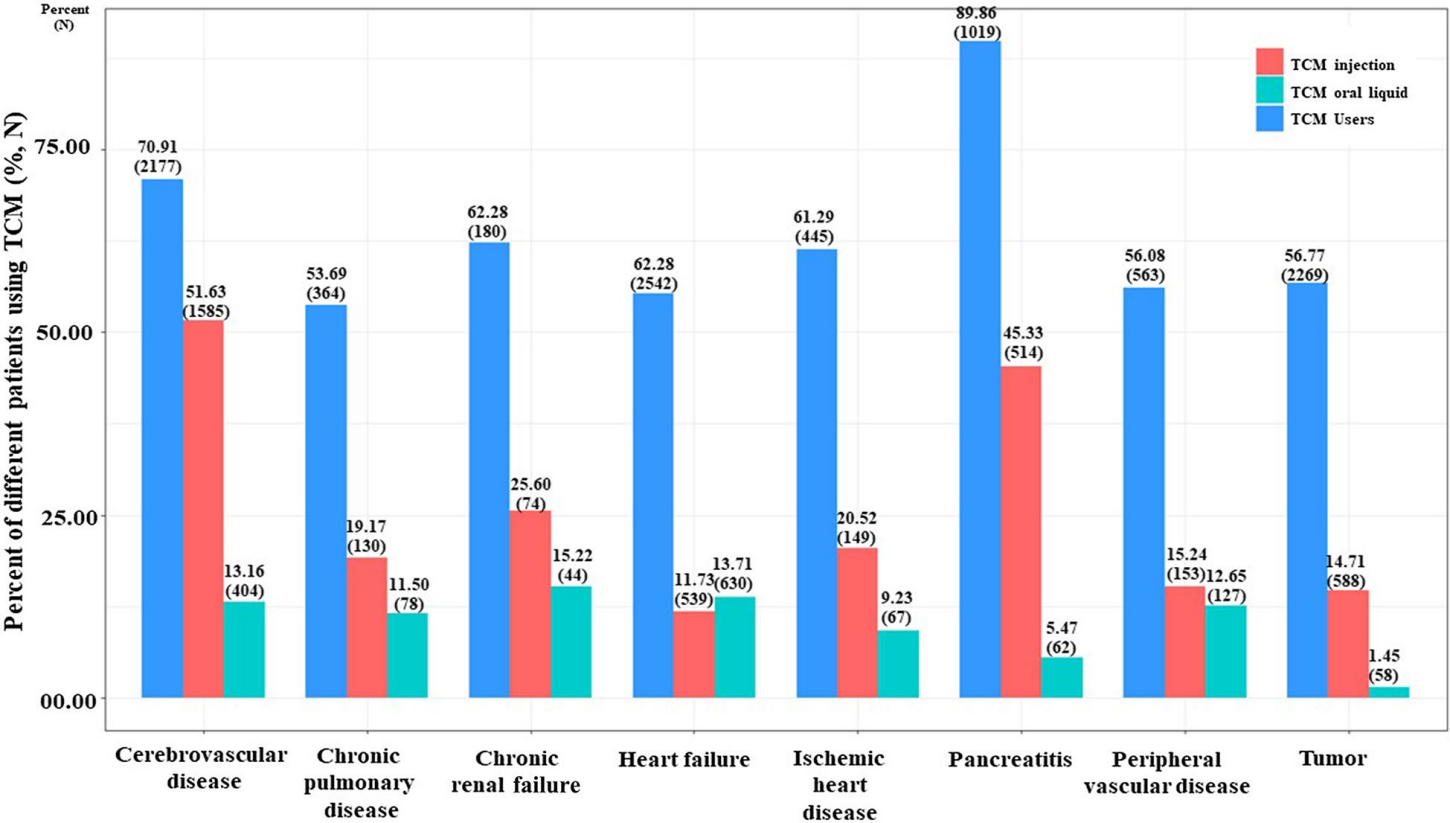
The utilization of different TCM types among ICU patients with comorbidity

The use of TCM interventions was associated with patient characteristics. Patients with pancreatitis (89.9%) were the population who used the most used TCM interventions, patients with cerebrovascular disease (51.6%) with the most TCM injections, and those with chronic renal failure (15.2%) with the most oral liquids (Fig. 1). Across eight patient populations with different comorbidities, Bupleurum injection, Tanreqing injection and Myrtol standardized enteric capsules were the most used TCM injection and oral liquid (Additional file 1: Table S5).

Our study suggested that TCM was commonly used among ICU patients and TCM injections were more commonly used, particularly in patients at neurological ICU. Our study was consistent with previous publication that suggested the wide use of TCMs in patients with cerebrovascular disease [13]. The wide use of TCM injections was partly due to the nature of the ICU care; in addition, availability of reimbursement for these prescriptions may support their clinical use [14]. We also found that the use of TCM was often associated with patient characteristics. For example, TCMs were mostly used in patients with pancreatitis, which might be related to the published guidelines for pancreatitis treatment in China [15]. TCM injections were mostly used for patients with cerebrovascular diseases, likely due to their advantages in this patient population. Our finding was consistent with a previous study [16].

To the best of our knowledge, this was the first study that investigated the treatment patterns of TCMs among ICU patients. Our study included a large number of patients from a well-established registry. However, this study has a few limitations. Firstly, information regarding herbal formulas (multiherb products) was unavailable, for which we were unable to look into details about their uses. Secondly, our study was based on data from a single health care institution, which may limit the generalizability of findings. Nevertheless, it is the largest medical center in west China that has the largest ICU departments in the region. Thus, the findings may represent the treatment patterns from the west China region.

To further rationalize the use of TCM in ICU, more high-quality evidence about clinical effects of TCM is warranted. In particular, efforts are needed to further improve the quality of clinical studies about TCM in ICU, and trustworthy, normative and operationalizable guidelines of TCM in ICU that are based on systematic review evidence and GRADE approach should be developed. In addition, wider application of TCM in the ICU patient would also largely rely on healthcare policy. As such, industry standards and norms that meet the features of TCM interventions in the ICU should be also formulated.

## Conclusions

Using data from a well-established registry, our study has clearly suggested that TCMs are commonly used among ICU patients in China. In particular, TCM injections and oral liquids are more often used than CHM. The use of TCM was also associated with patient characteristics and patients with pancreatitis were prescribed with most TCM. Future efforts should include systematic development of clinical evidence, guidelines and standards about clinical effects of TCM interventions in the ICU patients.

## Supplementary Information

**Additional file 1: Table S1.** Demographic characteristics of user or non-user of TCM. **Table S2.** The combinations use of TCM interventions among ICU patients. **Table S3.** The mostly used TCMs in the ICU. **Table S4.** The utilization of TCM among the ICU patients. **Table S5.** Mostly used TCM injections and TCM oral liquids among ICU patients with comorbidities.

## Data Availability

The datasets used and analysed during the current study are available from the corresponding author upon reasonable request.

## Abbreviations

CHM: Chinese herbal medicine
COVID-19: Coronavirus disease 2019
EMR: Electronic medical record
ICU: Intensive care unit
ICU-HAI: Healthcare-Associated Infections monitoring system in intensive care unit
ICD-10: International Classification of Diseases, 10th edition
NHC: National Health Commission
TCM: Traditional Chinese medicine
WCH: West China Hospital
