# Supplementary material for: Utilization of traditional Chinese medicine in the intensive care unit
Source: Chin Med. 2021 Aug 23;16:84. doi: 10.1186/s13020-021-00496-1 (PMC8382104; doi:10.1186/s13020-021-00496-1)
Supplement: Supplementary file 1 — Additional file 1: Table S1. Demographic characteristics of user or non-user of TCM. Table S2. The combinations use of TCM interventions among ICU patients. Table S3. The mostly used TCMs in the ICU. Table S4. The utilization of TCM among the ICU patients. Table S5. Mostly used TCM injections and TCM oral liquids among ICU patients with comorbidities. [file 13020_2021_496_MOESM1_ESM.doc]

**Table S1. Demographic characteristics of user or non-user of TCM**

| **Variables** | **TCM Users**  **(N=6583)** | **Non-TCM Users**  **(N=15760)** | ***P* value** |
| --- | --- | --- | --- |
| Age (Median, IQR) | 54 (45,66) | 55 (45,66) | 0.002 |
| Age (N, %) |  |  | 0.000 |
| 18-44 | 1625(24.68) | 3330(21.13) |  |
| 45-64 | 3018(45.85) | 7789(49.42) |  |
| 65-74 | 1200(18.23) | 2953(18.74) |  |
| > 75 | 740(11.24) | 1688(10.71) |  |
| Male (N, %) | 3900(59.24) | 9239(58.62) | 0.390 |
| APACHE II score (Median, IQR) | 16 (11, 21) | 15(10, 20) | 0.000 |

Abbreviations: TCM, Traditional Chinese Medicine; IQR, interquartile range; APACHE, Acute Physiology and Chronic Health Evaluation

Note: Chronic comorbidities included tumor, peripheral vascular disease, pancreatitis, ischemic heart disease, heart failure, chronic pulmonary disease, chronic renal failure and cerebrovascular disease.

**Table S2. The combinations use of TCM interventions among** ICU patients

| **Combinations** | **Total**  **(N=1881) (%)** | **Two medicines (N=1318) (%)** | **More than two medicines (N=563) (%)** | ***P* value** |
| --- | --- | --- | --- | --- |
| TCM injection + TCM injection | 1336 (71.08) | 1025 (77.84) | 311 (55.24) | <0.001 |
| TCM oral liquid + TCM oral liquid | 113 (6.00) | 98 (7.44) | 15 (2.66) |  |
| TCM oral liquid + TCM injection | 781 (41.52) | 455 (34.52) | 326 (57.90) |  |

Abbreviations: TCM, Traditional Chinese Medicine; ICU, intensive care unit

**Table S3. The mostly used TCMs in the ICU.**

| **TCMs** | **Number of**  **TCM Users** | **Proportion in ICU population (%)** |
| --- | --- | --- |
| TCM injection | 5424 | 24.28 |
| Bupleurum chinense DC | 3008 | 13.46 |
| Tanreqing injection | 2374 | 10.63 |
| Erigeron breviscapus injection | 829 | 3.71 |
| Shenfu Injection | 611 | 2.73 |
| Compound Glycyrrhizin Injection | 162 | 0.73 |
| TCM Oral Liquid | 1740 | 7.79 |
| Myrtol standardized enteric capsules | 668 | 2.99 |
| Fructus Cannabis Bolus | 592 | 2.65 |
| Changtai mixture | 184 | 0.82 |
| Zicao oil | 151 | 0.68 |
| Berberine hydrochloride | 80 | 0.36 |
| CHM | 790 | 3.54 |
| Jiang Magnolia | 726 | 3.25 |
| Radix Aucklandiae | 704 | 3.15 |
| Rhubarb | 698 | 3.12 |
| Mirabilite | 672 | 3.01 |
| Wine Radix Scutellariae | 666 | 2.98 |

Abbreviations: TCM, Traditional Chinese Medicine; ICU, intensive care unit;.CHM, Chinese herb medicine

**Table S4**. The utilization of TCM among the ICU patients.

| **Variables** | **TCM Users**  **(N=6583)** | **TCM injection**  **(N=5424)** | **TCM oral liquid**  **(N=1740)** | **CHM**  **(N=790)** |
| --- | --- | --- | --- | --- |
| ICU Ward (N, %) |  |  |  |  |
| GICU (N=5313) | 1455(27.39) | 1188(22.36) | 360(6.78) | 383(7.21) |
| RICU (N=1582) | 418(26.42) | 328(20.73) | 119(7.52) | 23(1.45) |
| SICU (N=5359) | 2162(40.34) | 2084(38.89) | 243(4.53) | 221(4.12) |
| TICU (N=7744) | 1182(15.26) | 579(7.48) | 702(9.07) | 9(0.12) |
| NICU (N=2345) | 1366(58.25) | 1245(53.09) | 316(13.48) | 154(6.57) |
| Years (N, %) |  |  |  |  |
| 2015 (N=4928) | 1858(37.70) | 1364(27.68) | 707(14.35) | 170(3.45) |
| 2016 (N=6024) | 1860(30.88) | 1557(25.85) | 480(7.97) | 166(2.76) |
| 2017 (N=5827) | 1670(28.66) | 1468(25.19) | 391(6.71) | 251(4.31) |
| 2018 (N=5615) | 1194(21.26) | 1034(18.41) | 161(2.87) | 203(3.62) |

Abbreviations: TCM, Traditional Chinese Medicine; CHM, Chinese herb medicine; ICU, intensive care unit; GICU, general intensive care unit; RICU, respiratory intensive care unit; SICU, Surgical intensive care unit; TICU, rhoracic surgery intensive care unit; NICU, neurological intensive care unit; PICU, pediatric intensive care unit

**Table S5. Mostly used TCM injections and TCM oral liquids among ICU patients with comorbidities**

| **Disease** | **TCM injection** | |  | **TCM oral liquid** | |
| --- | --- | --- | --- | --- | --- |
|  | **Name** | **Users (n, %)** |  | **Name** | **Users (n, %)** |
| Cerebrovascular disease (N=3070) | Tanreqing injection | 890 (28.99) |  | Fructus Cannabis Bolus | 339 (11.04) |
|  | Bupleurum chinense DC | 509 (16.58) |  | Myrtol standardized enteric capsules | 18 (0.59) |
|  | Erigeron breviscapus injection | 137 (4.46) |  | Changtai mixture | 18 (0.59) |
|  | Shenfu Injection | 32 (1.04) |  | Zicao oil | 10 (0.33) |
|  | Compound Glycyrrhizin Injection | 9 (0.29) |  | Berberine hydrochloride | 4 (0.13) |
| Tumor (N=3997) | Tanreqing injection | 328 (8.21) |  | Fructus Cannabis Bolus | 15 (0.38) |
|  | Bupleurum chinense DC | 192 (4.80) |  | Zicao oil | 12 (0.30) |
|  | Erigeron breviscapus injection | 31 (0.78) |  | Myrtol standardized enteric capsules | 9 (0.23) |
|  | Shenfu Injection | 24 (0.60) |  | Berberine hydrochloride | 7 (0.18) |
|  | Compound Glycyrrhizin Injection | 5 (0.13) |  | Changtai mixture | 5 (0.13) |
| Heart Failure (N=4594) | Shenfu Injection | 274 (5.96) |  | Myrtol standardized enteric capsules | 493 (10.73) |
|  | Bupleurum chinense DC | 182 (3.96) |  | Changtai mixture | 56 (1.22) |
|  | Tanreqing injection | 52 (1.13) |  | Fructus Cannabis Bolus | 35 (0.76) |
|  | Erigeron breviscapus injection | 11 (0.24) |  | Berberine hydrochloride | 13 (0.28) |
|  | Compound Glycyrrhizin Injection | 10 (0.22) |  | Zicao oil | 12 (0.26) |
| Pancreatitis (N=1134) | Bupleurum chinense DC | 286 (25.22) |  | Zicao oil | 18 (1.59) |
|  | Tanreqing injection | 147 (12.96) |  | Liuhe dan | 16 (1.41) |
|  | Shenfu Injection | 36 (3.17) |  | Fructus Cannabis Bolus | 11 (0.97) |
|  | Sodium Tanshinone IIA Sulfonate injection | 25 (2.20) |  | Compound Glycyrrhiza Oral Solution | 5 (0.44) |
|  | Erigeron breviscapus injection | 14 (1.23) |  | Yunnan Baiyao | 3 (0.26) |
| Peripheral vascular disease (N=1004) | Bupleurum chinense DC | 81 (8.07) |  | Myrtol standardized enteric capsules | 83 (8.27) |
|  | Shenfu Injection | 45 (4.48) |  | Changtai mixture | 13 (1.29) |
|  | Tanreqing injection | 15 (1.49) |  | Fructus Cannabis Bolus | 10 (1.00) |
|  | Erigeron breviscapus injection | 5 (0.50) |  | Berberine hydrochloride | 7 (0.70) |
|  | Compound Glycyrrhizin Injection | 4 (0.40) |  | Zicao oil | 4 (0.40) |
| Ischemic Heart Disease (N=726) | Tanreqing injection | 55 (7.58) |  | Myrtol standardized enteric capsules | 26 (3.58) |
|  | Bupleurum chinense DC | 55 (7.58) |  | Fructus Cannabis Bolus | 20 (2.75) |
|  | Erigeron breviscapus injection | 19 (2.62) |  | Changtai mixture | 7 (0.96) |
|  | Shenfu Injection | 15 (2.07) |  | Zicao oil | 6 (0.83) |
|  | Sodium Tanshinone IIA Sulfonate injection | 3 (0.41) |  | Bailing capsules | 4 (0.55) |
| Chronic pulmonary disease (N=678) | Bupleurum chinense DC | 78 (11.50) |  | Myrtol standardized enteric capsules | 25 (3.69) |
|  | Tanreqing injection | 31 (4.57) |  | Fructus Cannabis Bolus | 19 (2.80) |
|  | Erigeron breviscapus injection | 6 (0.88) |  | Changtai mixture | 10 (1.47) |
|  | Shenfu Injection | 6 (0.88) |  | Zicao oil | 8 (1.18) |
|  | Compound Glycyrrhizin Injection | 5 (0.74) |  | Burnet root leukopoietic tablets | 5 (0.74) |
| Chronic Renal Failure (N=289) | Bupleurum chinense DC | 45 (15.57) |  | Bailing capsules | 12 (4.15) |
|  | Tanreqing injection | 19 (6.57) |  | Fructus Cannabis Bolus | 12 (4.15) |
|  | Compound Glycyrrhizin Injection | 6 (2.08) |  | Myrtol standardized enteric capsules | 7 (2.42) |
|  | Erigeron breviscapus injection | 2 (0.69) |  | Zicao oil | 7 (2.42) |
|  | Sodium Tanshinone IIA Sulfonate injection | 2 (0.69) |  | Changtai mixture | 2 (0.69) |

Abbreviations: TCM, Traditional Chinese Medicine; ICU, intensive care unit.
